# Glucometabolic-Related Genes as Diagnostic Biomarkers and Therapeutic Targets for Alzheimer's Disease and Type 2 Diabetes Mellitus: A Bioinformatics Analysis

**DOI:** 10.1155/2024/5200222

**Published:** 2024-04-02

**Authors:** Shuo Liu, He Chen, Xiao-Dong He, Xiao-Ou Yang

**Affiliations:** The Fourth People's Hospital of Shenyang, Shenyang, Liaoning Province, China

## Abstract

**Background:**

Alzheimer's disease (AD) and type 2 diabetes mellitus (T2DM) are two widespread chronic disorders characterized by shared risk factors and molecular pathways. Glucose metabolism, pivotal for cellular homeostasis and energy supply, plays a critical role in these diseases. Its disturbance has been linked to the pathogenesis of both AD and T2DM. However, a comprehensive investigation into the specific roles of glucometabolic genes in the onset and progression of AD and T2DM has yet to be conducted.

**Methods:**

By analyzing microarray datasets from the Gene Expression Omnibus (GEO) repository, we identified differentially expressed glucometabolic genes (DEGs) in AD and T2DM cohorts. A range of bioinformatics tools were employed for functional annotation, pathway enrichment, protein interaction network construction, module analysis, ROC curve assessment, correlation matrix construction, gene set enrichment analysis, and gene-drug interaction mapping of these DEGs. Key genes were further validated using quantitative real-time polymerase chain reaction (qRT-PCR) in AD and T2DM murine models.

**Results:**

Our investigation identified 41 glucometabolic-related DEGs, with six prominent genes (G6PD, PKM, ENO3, PFKL, PGD, and TALDO1) being common in both AD and T2DM cohorts. These genes play crucial roles in metabolic pathways including glycolysis, pentose phosphate pathway, and amino sugar metabolism. Their diagnostic potential was highlighted by area under curve (AUC) values exceeding 0.6 for AD and 0.8 for T2DM. Further analysis explored the interactions, pathway enrichments, regulatory mechanisms, and potential drug interactions of these key genes. In the AD murine model, quantitative real-time polymerase chain reaction (qRT-PCR) analysis revealed significant upregulation of G6pd, Eno3, and Taldo1. Similarly, in the T2DM murine model, elevated expression levels of G6pd, Pfkl, Eno3, and Pgd were observed.

**Conclusion:**

Our rigorous research sheds light on the molecular interconnections between AD and T2DM from a glucometabolic perspective, revealing new opportunities for pharmacological innovation and therapeutic approaches. This study appears to be the first to extensively investigate glucometabolic-associated DEGs and key genes in both AD and T2DM, utilizing multiple datasets. These insights are set to enhance our understanding of the complex pathophysiology underlying these widespread chronic diseases.

## 1. Introduction

Alzheimer's disease (AD) and type 2 diabetes mellitus (T2DM) are two prevalent chronic diseases that affect millions worldwide [1]. AD, a progressive neurodegenerative disorder, is characterized by cognitive impairment, memory loss, and behavioral changes [2]. Its early symptoms result from synaptic dysfunction, which disrupts neural circuit connections, leading to continuous memory loss [3]. Research indicates that glucose, the primary energy source for brain activity, shows impaired metabolism in the brains of AD patients before structural changes and cognitive impairment become evident. This impairment in glucose metabolism may serve as an early indicator of AD [4].

T2DM, a metabolic disorder, is characterized by hyperglycemia, insulin resistance, and impaired insulin secretion [5]. This metabolic disturbance also increases the risk for dementia [6]. AD and T2DM share several metabolic defects, including insulin resistance, impaired glucose metabolism, and mitochondrial dysfunction [7]. Furthermore, T2DM has been linked to an increased risk of dementia [8, 9] and of AD by 45–90% [10, 11], as well as an increased risk of AD in T2DM patients [12]. Certain diabetes complications, such as imbalances between glucose and insulin, significantly elevate the risk of developing dementia [8].

The molecular mechanisms connecting AD and T2DM remain incompletely understood; however, accumulating evidence points to a critical role for glucose metabolism in both conditions [13]. Essential for maintaining cellular homeostasis and energy supply, glucose metabolism encompasses pathways like glycolysis, gluconeogenesis, the PPP, and glycogen synthesis/degradation [14]. Disruption in glucose metabolism can lead to the accumulation of toxic metabolites or intermediates, mitochondrial dysfunction, increased oxidative stress, and inflammation [15]. These pathological processes can compromise brain function and structure in AD patients or pancreatic *β*-cell function and insulin sensitivity in T2DM patients [16].

Glucose metabolism-related genes encode enzymes or regulators that modulate glucose metabolism. Alterations in these genes can impact metabolic pathways and disease pathogenesis [17]. Previous studies have identified associations between certain glucose metabolism-related genes and susceptibility or progression of AD or T2DM [18–20]. However, a comprehensive analysis of these genes in both AD and T2DM has yet to be conducted.

Given the rising global incidence of diabetes and neurodegenerative diseases, identifying new biomarkers associated with these conditions is critically important for developing future diagnostic methods. In our study, we conducted a thorough analysis of DEGs related to glucose metabolism in AD and T2DM using multiple datasets. We pinpointed six hub genes involved in key metabolic pathways with potential diagnostic relevance for both diseases. Our results contribute to a deeper understanding of the pathophysiology of these prevalent chronic diseases from a glucometabolic standpoint and offer novel targets for drug development and therapy.

## 2. Materials and Methods

### 2.1. Data Source

The purpose of this study is to study the biomarkers and therapeutic targets of AD and T2DM based on bioinformatics methods. Microarray expression data for AD and T2DM were downloaded from the Gene Expression Omnibus (GEO) database, with our methodologies informed by prior research [21]. A total of 104 control and 145 AD samples were sourced from the GSE63060 dataset which is a human blood sample-related AD, and the data are also matched in age and gender. The GSE95849 dataset is a human peripheral blood mononuclear cell sample of T2DM, including 6 T2DM samples and 6 control samples. These two datasets are in line with our research purposes, so these two datasets are selected.

The glucometabolic-related genes were obtained from the Gene Ontology-biological process (GO-BP) gene sets in the Molecular Signature Database (MSigDB; https://www.gsea-msigdb.org/gsea/msigdb/index.jsp).

### 2.2. Acquirement of Differentially Expressed Genes (DEGs)

Generally, all the microarray data after normalization were analyzed by R software.

R package “limma” was used to identify differentially expressed mRNAs in AD and T2DM dataset. The threshold of DEGs' screening was ∣log2FC| ≥ 1 and adj.*P*. Val <0.05 [22]. The DEGs obtained from the two datasets were visualized using the R packages “pheatmap” and “ggplot2” to generate the heat maps and volcano maps, respectively.

### 2.3. Functional Annotation and Pathway Enrichment Analysis

We overlapped DEGs from two datasets and glucometabolic-related genes to obtain glucometabolic-related DEGs both in AD and T2DM datasets.

To reveal the functions of DEGs, GO annotation [23] and Kyoto Encyclopedia of Genes and Genomes (KEGG) enrichment [24] analysis were conducted using the “clusterProfiler” package. The GO terms were composed of the following three categories: biological process (BP), cellular component (CC), and molecular function (MF). While the KEGG pathway enrichment analysis is prone to describe gene function in the genomic and molecular levels and show the correlated genes. Adj.*P*. Val <0.05 was regarded as statistically significant.

### 2.4. Protein-Protein Interaction (PPI) Network Construction

The PPI network was constructed through the Search Tool for the Retrieval of Interacting Genes (STRING) database [25]. Cytoscape software was used to visualize the network [26]. Then, Cytoscape plugin-MCODE was used to screen the significant modules in the PPI network.

### 2.5. Evaluation of Diagnostic Performance of Hub Genes

ROC curve analysis was performed using the pROC package to evaluate the diagnostic value of the hub genes for AD and T2DM, respectively. Receiver operating characteristic (ROC) curve analysis, which yields indictors of accuracy such as the area under the curve (AUC), provides the basic principle and rationale for distinguishing between the specificity and sensitivity of diagnostic performance [27].

### 2.6. Correlation Analysis and Gene Set Enrichment Analysis (GSEA) of Hub Genes

The “ggpubr” package was used to perform Spearman correlation analysis on hub genes. Moreover, GSEA of hub genes was performed using the “GSEABase” packages, and KEGG gene sets were used as a reference, with *P* < 0.05 being a statistically significant difference.

### 2.7. Construction of Gene-Drug Interaction Network and Regulatory Network of Hub Genes

In order to explore the potential therapeutic drugs for gene-related diseases, the targeted drugs of proteins encoded by key genes through the DGIDB database were identified (https://dgidb.genome.wustl.edu/) [28].

We used miRNet [29] databases to predict the TFs and miRNAs of hub genes. Hub genes and their TFs and miRNAs were integrated into a regulatory network and visualized using Cytoscape software.

### 2.8. Experimental Animals

The study, sanctioned by the Animal Experiment Committee of Shenyang Fourth People's Hospital, utilized male C57BL/6J mice (8 weeks old) sourced from China Medical University. The mice were maintained at a consistent temperature of 22 ± 2°C and subjected to a 12 : 12-hour light-dark cycle, with unrestricted access to food and water. Body weight and food intake were recorded weekly.

#### 2.8.1. Alzheimer's Disease (AD) Model

For the AD model, male 5XFAD transgenic mice from Jackson Laboratory, harboring familial AD mutations (K670N/M671L, I716V, V717I in *App*; M146L, L286V in *Ps1*) [30], along with wild-type C57BL/6J mice, were utilized for AD and control groups, respectively (8 mice/group). Genotyping was performed using PCR from tail DNA samples.

#### 2.8.2. Type 2 Diabetes Mellitus (T2DM) Model

The T2DM model involved inducing the condition in male C57BL/6J mice using streptozotocin (STZ, 55 mg/kg; Solarbio, batch S0130) [31]. The mice were categorized into STZ-treated and control groups (8 mice/group), with the former receiving daily intraperitoneal injections of STZ for 5 consecutive days. Blood glucose levels were monitored four weeks postinjection using an ACCU-CHEK glucometer (Roche Diagnostics).

Blood samples were collected from all mice at 12 weeks for analysis. The bleeding techniques and administration of anesthetics and analgesics adhered to contemporary veterinary guidelines. All mice were treatment-naïve prior to this study. For all procedures, approximately 100 *μ*l of blood was collected using either marked capillary pipettes or a syringe and immediately stored in EDTA-coated tubes (BD Microtainer) for thorough mixing. Experienced researchers conducted all bleeding in this study.

A modified tail-clip procedure was employed [32]. Briefly, 2.5% Lidocaine and 2.5% Prilocaine (EMLA) cream (Hi-Tech Pharmacal Co., Inc, Amityville, NY) was topically applied to the distal 0.5 cm of the tail 15 minutes prior to bleeding. A segment of ≤1 mm was amputated with surgical scissors, and blood was collected using capillary pipettes.

### 2.9. Quantitative Real-Time Polymerase Chain Reaction (qRT-PCR)

Total RNA was extracted from the peripheral blood of mice using TRIzol® reagent (Invitrogen Life Technologies, Carlsbad, CA, United States). Reverse transcription of total RNA was performed using a high-capacity cDNA reverse transcription kit (code: FSQ-101, Osaka, Japan). The primer sequences for qRT-PCR are as follows for *G6pd* primers: 5′-CAG GGA CGA GCT CCT TGA G-3′ and 5′-GGG GGT TCA CCC ACT TGT AG-3′; *Eno3* primers: 5′-CGC AGA TCT TGC AGG CAA TC-3′ and 5′-GGG TCA TCG GGT GAC TTG AA-3′; *Taldo1* primers: 5′-AAA AAG TTG GCA TGT CGA GC-3′ and 5′-GCT GAT CCC AGC TTC CTT GT-3′; *Pfkl* primers: 5′-TCA TGT GTG TCA TCC CAG CC-3′ and 5′-CAT GCG GTG CTC GAA ATC AG-3′; *Pgd* primers: 5′-CCA TGG CCC AAG CTG ACA TC-3′ and 5′-ACC GTC TTG TGG TGT CCC TA-3′. For qRT-PCR, SYBR Green PCR Master Mix (Cat. No. 04913850001, Roche, Germany) and the BIO-GENER Real-Time System (China) were used according to the manufacturer's instructions. The number of mice in each group was eight, and each sample was tested in triplicate-independent qRT-PCR. The threshold cycle (Ct) values were standardized to the glyceraldehyde-3-phosphate dehydrogenase (GAPDH) values measured on the same plate, and the 2^−ΔΔCt^ method ·was used to determine the fold changes in gene expression [33].

### 2.10. Statistical Analysis

In this study, R software was used for statistical analysis. Spearman correlation analysis was used to evaluate the correlation between continuous variables. The difference of variables between the disease group and the control group was evaluated by the Wilcoxon rank sum test, and GraphPad Prism 9.0 was used for statistical analysis and visualization of qRT-PCR. ^*∗*^*P* < 0.05, ^*∗∗*^*P* < 0.01, and ^*∗∗∗*^*P* < 0.001. *P* < 0.05 was considered statistically significant, and the calibration method of *P* value is the false discovery rate (FDR).

## 3. Results

### 3.1. Identification of Differentially Expressed Glucometabolic-Related Genes and Functional Analysis

**Figure 1:**
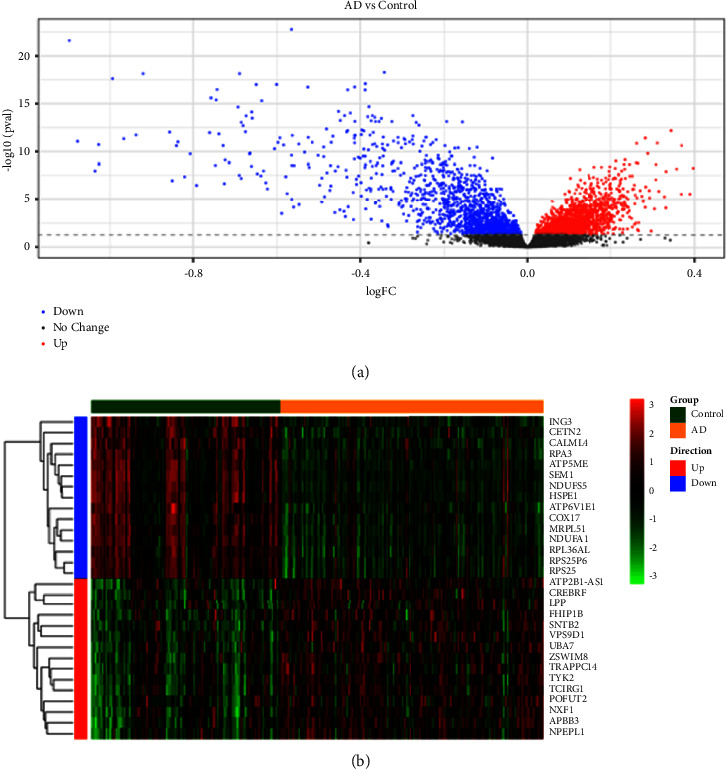
Identification of differentially expressed genes (DEGs) in the GSE63060 dataset. (a) The volcano plot of DEGs in the GSE63060 dataset, including 1652 upregulated genes and 1496 downregulated genes. (b) The heatmap of DEGs in the GSE63060 dataset for the top 15 upregulated and top 15 downregulated DEGs.

A total of 3148 DEGs, including 1652 up-regulated genes and 1496 down-regulated genes, were identified in the AD dataset GSE63060. The volcano map of DEGs is shown in Figure 1(a). The heatmap for the top 15 upregulated and top 15 downregulated DEGs is displayed in Figure 1(b).

**Figure 2:**
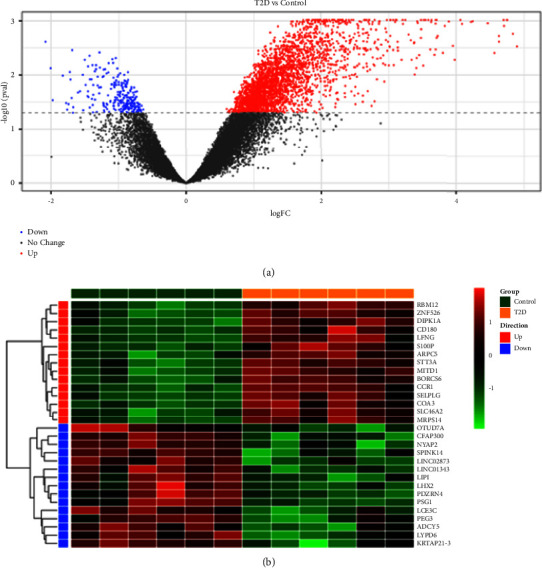
Identification of DEGs in the GSE95849 database. (a) The volcano plot of DEGs in the GSE95849 dataset, including 3023 upregulated genes and 257 downregulated genes. (b) The heatmap of DEGs in the GSE95849 dataset for the top 15 upregulated and top 15 downregulated DEGs.

A total of 3280 DEGs were identified in the T2DM dataset GSE95849. Of these DEGs, 3023 genes were upregulated and 257 genes were downregulated. The volcano map of DEGs is shown in Figure 2(a). The heatmap for the top 15 upregulated and top 15 downregulated DEGs is displayed in Figure 2(b).

**Figure 3:**
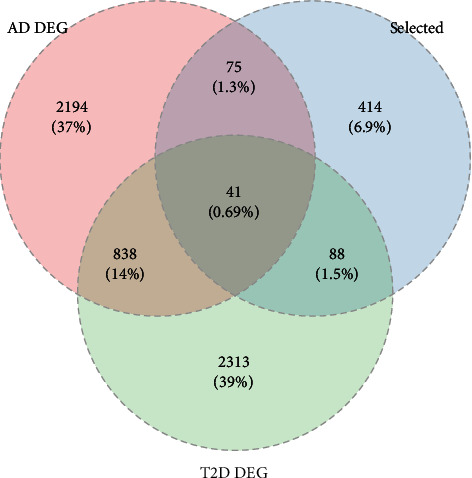
The Venn map of 41 glucometabolic-related DEGs between Alzheimer's disease (AD) DEGs, type 2 diabetes mellitus (T2DM) DEGs, and glucometabolic-related genes.

**Figure 4:**
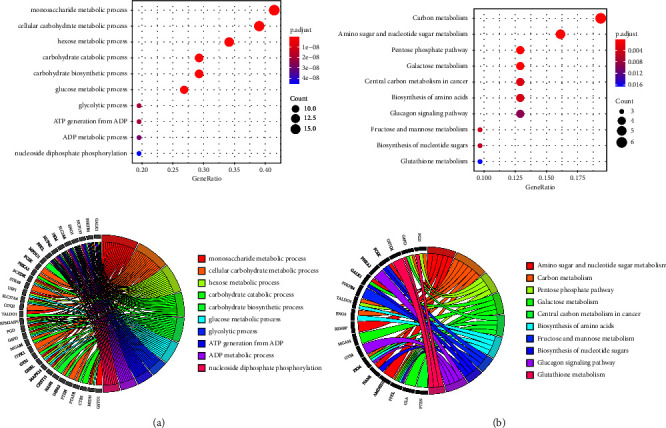
Functional analysis of 41 glucometabolic-related DEGs. (a) Gene Ontology (GO) enrichment analysis of 41 glucometabolic-related DEGs. (b) Kyoto Encyclopedia of Genes and Genomes (KEGG) enrichment analysis of 41 glucometabolic-related DEGs. Adjusted *P* value <0.05 was considered significant.

After intersecting the glucometabolic-related genes with DEGs from two datasets, we found total 41 glucometabolic-related DEGs (Figure 3). GO analysis revealed that 41 glucometabolic-related DEGs were enriched in the monosaccharide metabolic process, cellular carbohydrate metabolic process, and hexose metabolic process (Figure 4(a)). Analysis of the KEGG signal pathways revealed that glucometabolic-related DEGs were mainly enriched in carbon metabolic, amino sugar and nucleotide sugar metabolism, and pentose phosphate pathway (Figure 4(b)).

### 3.2. PPI Network Construction and Module Analysis

**Figure 5:**
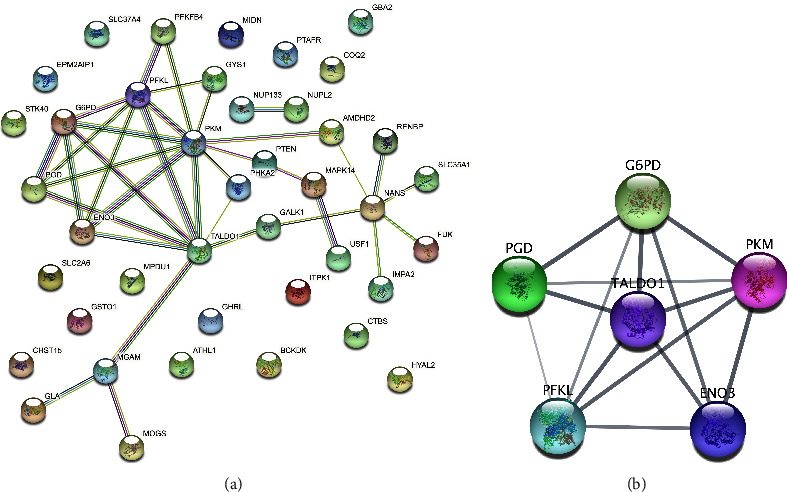
Construction of the protein-protein interaction (PPI) network. (a) PPI network of candidate genes consisting of 6 genes and 14 edges. (b) PPI network of six potential hub genes.

To further study the interaction of 41 glucometabolic-related DEGs, we constructed the PPI network by using the STRING database (Figure 5(a)). The most significant module consisting of 6 genes and 14 edges was found using the MCODE plug-in of Cytoscape software. Further, six genes including *G6PD*, *PKM*, *ENO3*, *PFKL*, *PGD*, and *TALDO1* in the key module were selected as potential hub genes (Figure 5(b)).

### 3.3. Evaluation of Diagnostic Performance of Hub Genes

**Figure 6:**
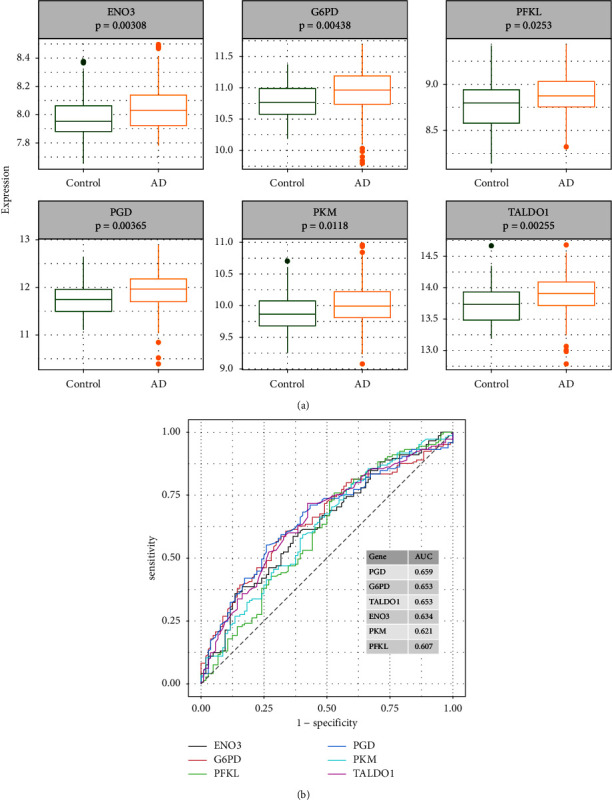
Analysis of six hub gene expression in AD patients in the GSE63060 dataset. (a) The expression level of six hub genes. (b) ROC curve of six hub genes.

**Figure 7:**
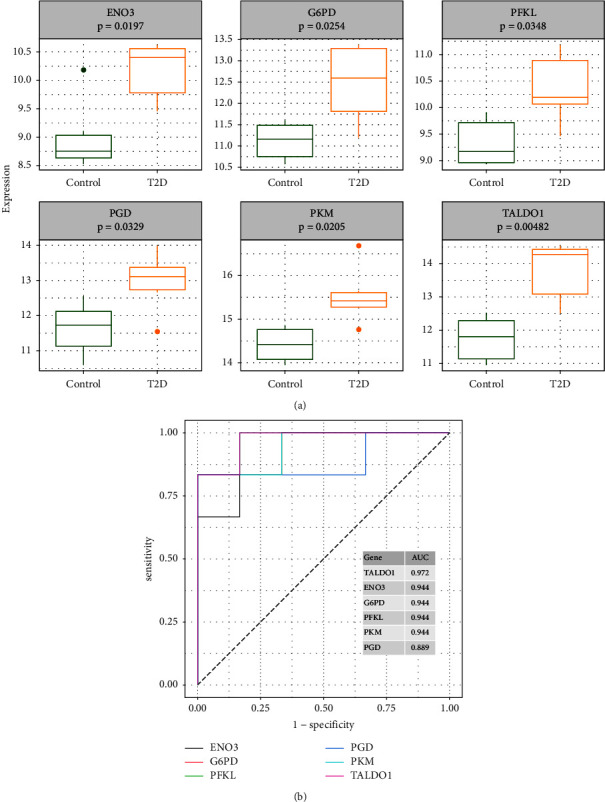
Analysis of six hub gene expression in T2DM patients in the GSE95849 dataset. (a) The expression level of six hub genes. (b) ROC curve of six hub genes.

We analyzed the expression level of hub genes in AD and T2DM, respectively. The results showed that the expression of all hub genes was upregulated in AD patients in GSE63060 (Figure 6(a)). For ROC curve analysis, the AUC values of the six hub genes were above at 0.6 (Figure 6(b)). In addition, all hub genes were upregulated in T2DM patients in GSE95849 (Figure 7(a)). For ROC curve analysis, the AUC values of the six hub genes were above at 0.8 (Figure 7(b)), which indicated that these genes had better distinguish performance in T2DM.

### 3.4. Correlation and GSEA Analyses for Hub Genes

**Figure 8:**
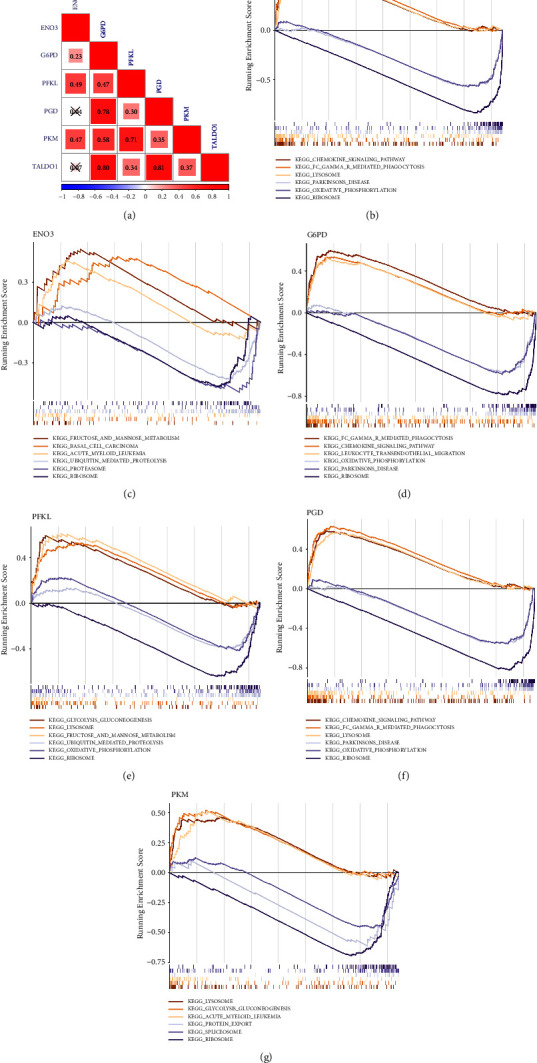
Correlation and gene set enrichment analysis (GSEA) analyses for hub genes in the GSE63060 dataset. (a) The correlation analysis of six hub gene expression. GSEA of six hub genes. *TALDO1* (b), *ENO3* (c), *G6PD* (d), *PFKL* (e), *PGD* (f), and *PKM* (g).

In AD, the correlation results showed that *TALDO1* was highly positively correlated with *PGD* and *G6PD* (Figure 8(a)). By performing GSEA analysis of each hub gene, we found that all hub genes were enriched in ribosome. Moreover, *G6PD*, *PFKL*, *PGD*, and *TALDO1* were also associated with oxidative phosphorylation. *PFKL*, *PGD*, *PKM*, and *TALDO1* were also associated with lysosome. *G6PD*, *PGD*, and *TALDO1* were also associated with Parkinson disease (Figures 8(b)–8(g)).

**Figure 9:**
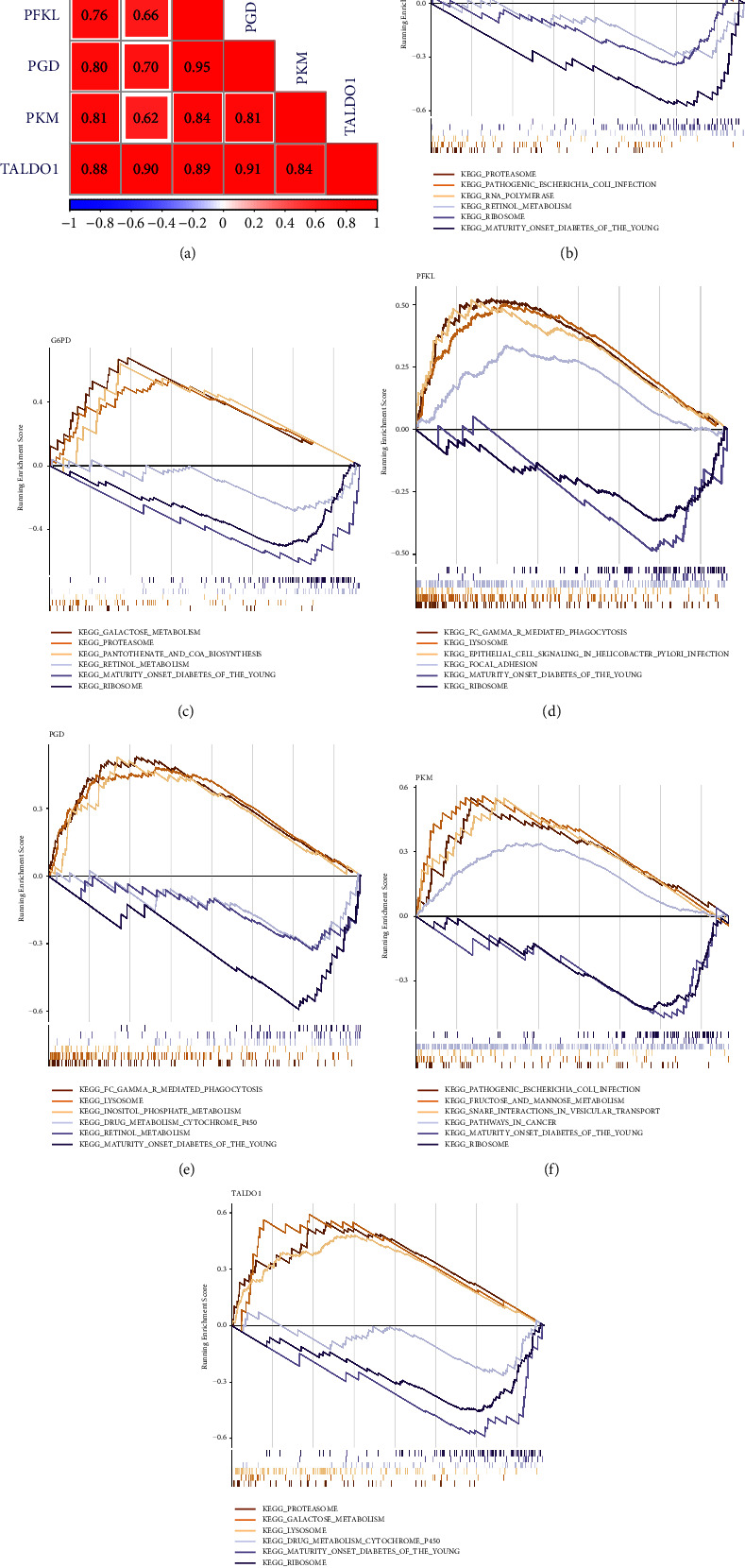
Correlation and GSEA analyses for hub genes in the GSE95849 dataset. (a) The correlation analysis of six hub gene expression. GSEA of six hub genes. *ENO3* (b), *G6PD* (c), *PFKL* (d), *PGD* (e), *PKM* (f), and *TALDO1* (g).

In T2DM, *PGD* and *PFKL* had the strongest correlation, followed by the correlation between *PGD* and *TALDO1* (Figure 9(a)). By performing GSEA analysis of each hub gene, we found that *ENO3*, *G6PD*, *TALDO1*, *PKM*, and *PFKL* were enriched in ribosome. In addition, *ENO3*, *G6PD*, and *TALDO1* were also associated with proteasome. *TALDO1* and *PGD* were also associated with lysosome (Figures 9(b)–9(g)).

### 3.5. Construction of Gene-Drug Interaction Network and Regulatory Network of Hub Genes

**Figure 10:**
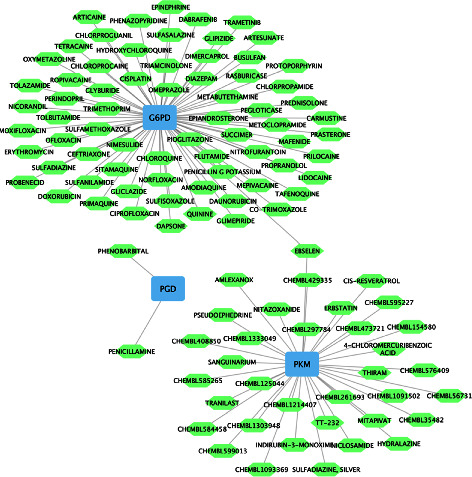
Construction of gene-drug interaction network. The potential therapeutic drugs for the diseases related to six hub genes.

In order to explore the potential therapeutic drugs for the diseases related to 6 hub genes, the compounds targeting the proteins encoded by hub genes were identified (Figure 10). Only three genes, *G6PD*, *PGD*, and *PKM*, were found to have interactions with small molecule drugs in the DGIdb database. There were 108 drugs with therapeutic effects on 3 hub genes, such as sulfamethoxazole, glyburide, and nitrofurantoin.

**Figure 11:**
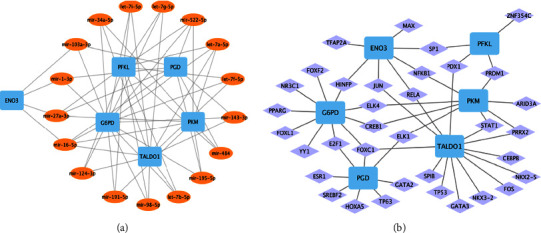
Construction of hub gene regulatory network. (a) Construction of miRNAs-hub gene network. The blue squares indicate hub genes, and the brown ovals indicate miRNAs. (b) Construction of TFs-hub gene network. The blue squares indicate hub genes, and the purple rhombus indicates TFs.

By using the miRNet database, the miRNAs-hub genes and TFs-hub genes networks were constructed by Cytoscape software (Figures 11(a) and 11(b)). Among them, miRNAs targeting at least three hub genes were selected to construct the network. Finally, the network was composed of 6 hub genes, and 17 miRNAs and 36 TFs were established. The blue squares were hub genes, the brown ovals were miRNAs, and the purple rhombus was TFs.

### 3.6. Validation of Hub Genes

**Figure 12:**
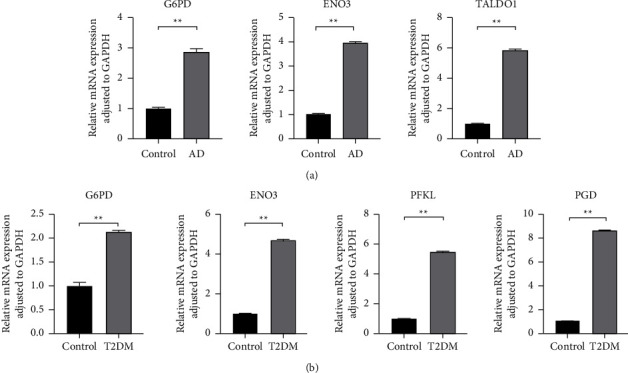
The expression of six hub genes by quantitative real-time PCR (qRT-PCR). (a) The expression of *G6pd*, *Eno3*, and *Taldo1* by qRT-PCR in the AD mice model compared to the control. ^*∗∗*^*P* < 0.01. (b) The expression of *G6pd*, *Eno3*, *Pfkl*, and *Pgd* by qRT-PCR in the T2DM mice model compared to the control. ^*∗∗*^*P* < 0.01.

To validate the results of the bioinformatics analysis, the expression of hub genes in AD, T2DM, and mouse peripheral blood was analyzed using qRT-PCR, and statistical analysis of the data results was performed using GraphPad Prism 9.0. Consistent with the bioinformatics results, it was found that, compared to normal mice in peripheral blood, AD mice exhibited increased expression of *G6pd*, *Eno3*, and *Taldo1* (Figure 12(a)). Similarly, T2DM mice showed increased expression of *G6pd*, *Pfkl*, *Eno3*, and *Pgd* (Figure 12(b)).

## 4. Discussion

Glucose metabolism is essential for maintaining cellular homeostasis and energy supply. Disorders in glucose metabolism are linked to various diseases, including AD and T2DM [34–36]. Abnormal glucose metabolism in the brain is now recognized as an early biomarker of AD and may also contribute to its pathogenesis, affecting A*β* and Tau metabolism, mitochondrial function, cellular signaling, and neuronal plasticity [13]. Increasing evidence indicates a connection between DM and AD with impaired glucose homeostasis and altered brain function [37]. Abnormal glucose metabolism thus acts as a link between DM and AD. The primary aim of our study was to identify the differentially expressed genes (DEGs) related to glucose metabolism abnormalities in AD and DM, uncover potential targets, and explore their shared possible pathogenesis.

In our research, we identified 41 glucose metabolism-related DEGs in AD and T2DM patients through bioinformatics analysis. We also pinpointed six central genes (*G6PD*, *PKM*, *ENO3*, *PFKL*, *PGD*, and *TALDO1*) involved in key metabolic pathways with diagnostic potential for both diseases. Moreover, we investigated the correlation, enrichment, regulation, and drug interactions of these hub genes.

In our study, DEG enrichment was observed in monosaccharide metabolism, cellular carbohydrate metabolism, and hexose metabolism. Abnormal cellular carbohydrate metabolism is a reliable biomarker for diagnosing precursor AD in MCI patients [38]. The main pathways in cellular carbohydrate metabolism include glycolysis and the pentose phosphate pathway (PPP). These pathways involve the participation and regulation of multiple enzymes. Notably, hub genes *G6PD*, *PKM*, *ENO3*, *PFKL*, *PGD*, and *TALDO1* are key enzymes in this study. *G6PD* and *ENO3* have been implicated in AD risk and progression [39, 40], and an increase in *G6PD* activity is related to T2DM [41].

We also assessed the diagnostic performance of these hub genes through ROC curve analysis, suggesting new possibilities for AD diagnosis. Unlike T2DM, where diagnosis primarily relies on laboratory markers, AD diagnosis is predominantly based on clinical practice. Blood-based biomarker detection offers a novel approach for early diagnosis and monitoring of AD. Detecting these genetic markers in blood samples during the disease's early stages could facilitate AD identification before clinical symptoms' manifest, potentially enhancing treatment efficacy and disease management. Biomarkers from peripheral blood samples are increasingly favored due to their noninvasiveness and cost-effectiveness compared to other methods, and they may reflect pathological changes in the brain [42, 43].

In this research, we predicted a total of 108 drugs with therapeutic effects on the *G6PD*, *PGD*, and *PKM* hub genes. Some of these drugs are reported to benefit AD while treating T2DM. For instance, Sulfonylureas, commonly used for diabetes treatment, have shown potential in influencing AD progression by affecting the central nervous system's potassium ATP channel, providing potential AD treatment strategies [44]. Furthermore, the potential link between AD and brain insulin resistance [45] has made insulin sensitizers like metformin a focus in AD treatment. A placebo-controlled clinical trial demonstrated that metformin improved learning, memory, and attention in AD patients [46]. There is substantial evidence of a strong connection and similar pathological mechanisms between AD and T2DM, with a growing number of studies exploring the therapeutic effects of antidiabetic drugs on AD [47, 48].

Additionally, we constructed a miRNAs-hub gene and TFs-hub gene regulatory network using the miRNet database. We discovered that 17 miRNAs target at least three hub genes, and 36 TFs regulate at least one hub gene. These miRNAs and TFs, including miR-9, miR-375, and miR-124a, are reported to be associated with T2DM, insulin resistance, and *β*-cell dysfunction pathogenesis [49, 50]. They may regulate the glucose metabolism pathways of AD and T2DM by influencing the expression of key enzymes such as *G6PD*, *PGD*, and *PKM*.

However, it is important to acknowledge some limitations of this study. Firstly, being a microarray data analysis, it is essentially retrospective research. While this approach expedites the identification of disease mechanisms, it necessitates further external validation to confirm our findings. Moreover, the study primarily focused on gene expression changes, not comprehensively considering other regulatory mechanisms like epigenetic regulation and protein posttranslational modifications. Also, although some diabetes drugs showed improvement in cognitive impairment in AD patients, inconsistent results have been observed in *in vivo* and clinical studies [51]. Their effects might depend on complex underlying pathological processes. Furthermore, while this study evaluated the diagnostic performance of key genes via ROC curve analysis, accurate blood biomarkers for AD diagnosis are still under investigation. The correlation between peripheral markers and brain changes is not fully established, and the accumulation of various pathological features in the brain is thought to initiate symptom onset. Additionally, in progressive diseases like AD, chronic comorbidities can cause numerous changes in markers, potentially reflected in peripheral blood concentrations [52]. Hence, caution is advised in interpreting blood sample results for central disease assessment.

In summary, our study identified six central genes (*G6PD*, *PKM*, *ENO3*, *PFKL*, *PGD*, and *TALDO1*) differentially expressed in AD and T2DM patients, which are involved in key glucose metabolism pathways. These hub genes hold promise as biomarkers for disease diagnosis and potential therapeutic targets.

## Data Availability

The microarray expression data for AD and T2DM were downloaded from the Gene Expression Omnibus (GEO) database (https://www.ncbi.nlm.nih.gov/geo/). The accession numbers are GSE63060 and GSE95849. The glucometabolic-related genes were obtained from the GO-BP gene sets in the Molecular Signature Database (MSigDB; https://www.gsea-msigdb.org/gsea/msigdb/index.jsp). The data generated during this study are available upon reasonable request from the corresponding author.
